# Supplementary material for: National medicines regulatory authorities financial sustainability in the East African Community
Source: PLoS One. 2020 Jul 23;15(7):e0236332. doi: 10.1371/journal.pone.0236332 (PMC7377437; doi:10.1371/journal.pone.0236332)
Supplement: S1 File — (PDF) [file pone.0236332.s001.pdf]

S1: QUESTIONNAIRE AND CHECKLIST FOR HEADS OF NMRAs & M&E EXPERTS

This detailed survey for NMRA provides an overview of the regulatory system in a country including existing capacities and limitations and participation in the East African Community medicines regulatory harmonization scheme. Please fill in the required information and place a tick or a cross in your answer to the questions below:

CATEGORY	YES	NO	N/A
**POLICY AND LEGAL FRAMEWORK**			
**Policy Framework**			
Is access to quality, safe and efficacious medicines one of the priority components of a national health delivery system?			
Is there a national medicines policy (NMP) in the country? (Yes or No)			
If Yes; when was the NMP approved by the Government? (Indicate year of approval)			
Does the NMP provide for establishment of an autonomous national medicines regulatory agency (NMRA)? (Yes or No)			
**Legal Framework**			
Is there a Law for regulating medicine in your country? (Yes or No)			
What is the name of the law regulating medicines in the country?<br>Mention……………………………………………………………………………<br>……………………….			
When was the medicines law enacted?<br>Indicate year: ……………………………………………………………….			
Indicate if the following provisions and functions are covered in the legislation			

Market Authorization			
Licensing Manufacturers, Importers, Exporters, Wholesalers and Distributors			
Post Market surveillance and safety monitoring			
Regulatory Inspection and Enforcement			
Control of Clinical Trials of Medical Products			
Control of Promotion and Advertisement of Medical Products			
Establishment of Quality Control Laboratory			
Scheduling, Classification and Control of medical products			
Prohibition of Substandard/Spurious/Falsified/Falsely-labelled/Counterfeit (SSFFC) medical products			
Control of narcotics and psychotropic substances			
Scope of regulated products			
Offences and legal proceedings			
Administrative appeal procedures			
International Cooperation and Harmonization of Regulation of Medical Products			
Monitoring and Evaluation of National Regulatory Systems			
Transparency and information sharing			
Monitoring and evaluation of regulatory systems			
Power to make regulations			
Number and type of Regulations made under the Act			

Specified Guidelines to be made under the Act			
Declaration and Conflict of interests			
Restriction of liability			
Protection of and access to information			
Regulation of other related products			
Commencement and entry into force			
**GOVERNANCE, MANAGEMENT AND FINANCING**			
NMRA Governance			
Is the NMRA autonomous? Yes of No			
Is there a Governing Board for NMRA? Yes or No			
If Yes, indicate if the Board is Strategic, Executive or Advisory in its nature of functions			
Are there Technical/Expert Committee(s) responsible for assessing applications for registration of pharmaceutical products and GMP inspection? (Yes or No).			
Who makes the final registration decision? (Tick as appropriate)	Yes	No	
Board			
Technical/Expert Committee			
Other (specify)			
Indicate frequency of Technical Committee and Board meetings per annum (p.a.)	<2 p.a.	2-4 p.a.	> 4 p.a.

Board			
Technical/Expert Committee on Registration of medicines			
Technical/Expert Committee on GMP Inspection			
NMRA Management			
Quality Management System (QMS) in EAC			
Has the NMRA instituted the EAC QMS (Yes or No)			
If Yes, is the NMRA registration processes ISO Certified (Yes or No)			
**Management Information System (MIS) in EAC**			
Has the NMRAs instituted the EAC-MIS (Yes or No)			
Is the NMRA sharing regulatory information with others in the EAC region (Yes or No)			
Is the NMRA MIS linked to other NMRAs in the EAC region (Yes or No)			
Is the NMRA MIS linked to EAC Secretariat (Yes or No)			
Has the NMRA instituted e-CTD for registration of medicines (Yes or No)			
**NMRA FINANCING**			
What is the total annual budget (in US$) for the NMRA and financing gap for the past five (5) years?			
2011			
2012			
2013			
2014			
2015			

What are the sources of funding for the NMRA in US$? If possible, please indicate the approximate % by source for the last 4 years.			
Government			
Industry Fees			
Donors			
Other (please specify)			
Is the regulatory authority allowed to retain service fees collected from industry for execution of regulatory functions? Yes/No/Partly.			
If Yes or Partly; provide explanation how is the money used:<br>…………………………………………………<br>…………………………………………………			
Is the NMRA Audited annually (Yes or No)			
If Yes, indicate audit outcome for the past five (5) years (e.g. unqualified, qualified or adverse opinion)			
2011			
2012			
2013			
2014			
2015			
