# Supplementary material for: National medicines regulatory authorities financial sustainability in the East African Community
Source: PLoS One. 2020 Jul 23;15(7):e0236332. doi: 10.1371/journal.pone.0236332 (PMC7377437; doi:10.1371/journal.pone.0236332)
Supplement: S2 File — (PDF) [file pone.0236332.s002.pdf]

S2: INDICATORS GUIDANCE NOTES

Guidance to indicators measurement

The matrix below provides indicator title, purpose, rationale for selecting the indicators, method of measurement[1], measurement frequency and unit of measurement, sources, indicator classification, data type and institution responsible for reporting on the respective indicators.

**CATEGORY 1: POLICY AND LEGAL FRAMEWORK**
Indicator 1: National Medicines Policy (NMP)1
**a) Purpose:** The indicator assesses whether the country has a current National Medicines policy (NMP) and if the available policy is comprehensive in line with WHO recommendations.
**b) Rationale:** National Medicine Policy expresses and prioritizes the medium- to long-term goals set by the government for the pharmaceutical sector, and identifies the main strategies for attaining them. The policy is aimed at promoting equity and sustainability of the pharmaceutical sector.<br><br>The general objectives of a national medicines policy are to ensure; I) access; ii) quality; and iii) rational use of medicines. Specifically, the policy ensures; equitable availability and affordability of essential medicines (access); the quality, safety and efficacy of all medicines (quality); the promotion of therapeutically sound and cost-effective use of medicines by health professionals and consumers (rational use); and increase the national pharmaceutical production capacity. A national medicines policy expresses the commitment to a goal and a guide for action.
**c) Method of measurement:** Availability, comprehensiveness and how current a policy[1] is will be determined responses three parts:<br>1) Availability of a National Medicines Policy (NMP)<br>2) Comprehensiveness of the NMP will focus on the following key components as recommended by WHO[1]:<br>3) Year of last review or amendment of the policy by the Cabinet of Ministers
**d) Evidence/ Sources:** WHO NRA GBT, Government gazette, Ministry of Health
**f) Institutional Responsibility:** Ministry of Health
**g) Measurement frequency:** Three yearly
**h) Unit of measurement:** Yes/ No
**i) Data Type:** Qualitative
**j) Indicator Classification:** Input
Indicator 2: Legal framework governing regulation of medical products1
**a) Purpose:** This indicator assesses whether a country has a legal provision governing regulation of medical products or not, and its comprehensiveness based on the AU Model law.

b) Rationale: One of the challenges in ensuring effective regulation of medical products in Africa is the existence of numerous gaps in legal provisions in Member States. Also, the existing legal provisions in most countries vary in comprehensiveness and do not have clauses enabling NMRAs to utilise decisions made by others in the region nor internationally. The gaps and differences in legislation are a major hurdle in harmonization at the regional level. The ***African Union Model Law on Medical Product Regulation*** thus provides a systematic approach in the review and development of national legislation that will enable Member States to undertake their obligation to protect the health of their people. The purpose of the African Union Model Law is to provide a comprehensive guide to countries as they review or develop national laws to facilitate regulatory systems strengthening and harmonisation.

c) Method of measurement: Availability of a Law for regulating medicine in your country is assessed by:

1) Availability of a Law for regulating medicine in your country and the year of enactment of medicines law

2) Comprehensiveness of the law will be assessed based on the following elements as defined in the African Union Model Law on Medical Product Regulation:

-

d) Evidence/ Sources: WHO NRA GBT, Government Gazette, National Law, Ministry of Justice library, NMRA records

e) Institutional Responsibility: NMRA, Ministry of Health

f) Measurement frequency: Three yearly

g) Unit of measurement: Yes/ No

h) Indicator Classification: Input

i) Data Type: Qualitative

CATEGORY 2: NMRA GOVERNANCE

Indicator 3: NMRA level of autonomy 1

a) Purpose: This indicator assesses the level of autonomy of an NMRA

b) Rationale: One of the key challenges on regulatory systems strengthening in most countries in Africa is the absence of an autonomous1 National Medicines Regulatory Authority mandated to regulate the market. In countries where regulatory functions are split among two or more agencies, there is usually duplication of efforts, lapses in implementation, inconsistencies and spreading of limited resources too thinly. The AU Model Law provides for the establishment of autonomous NMRAs for effective coordination and regulation of medical products in a country.

1) **Method of measurement:** NMRA level of autonomy is assessed by the level of autonomy of the NMRA indicating either of the following:

- Fully autonomous
- Semi-autonomous
- Department under the Ministry of Health

2) If autonomous, respondent indicate the following:

- *Ability to generate and utilise revenue*
- *NMRA decision making power*
- *Ability of NMRA to recruit staff*[1]
- *Ability of NMRA to sue or be sued*

**c) Evidence/ sources:** WHO NRA GBT, CIRS OpERA programme, Government gazette, NMRA Financial report, Governance structure
**d) Institutional Responsibility:** NMRA, Ministry of Health
**e) Measurement frequency:** Three yearly
**f) Unit of measurement:** Yes/ No
**g) Data Type:** Qualitative
**h) Indicator Classification:** Output
**CATEGORY 3: NMRA FINANCING**
Indicator 5: Level of NMRA funding
**a) Purpose:** The total funding means the money that has actually been received in a given year by the NMRA and includes all sources such as government funding, fees for services rendered, income from investments, partner funding etc.
**b) Rationale:** Limited funding for National Medicines Regulatory Authorities (NMRA) is one of the major challenges hampering regulatory systems in most African countries. Financial capacity of any NMRA has a direct impact on its performance and growth. Population is a proxy for the size of the pharmaceutical market which will determine the level of investment required to control the market. This indicator therefore contributes to the understanding of the ability of the NMRA to regulate the market.
**c) Method of measurement:** The indicator is calculated as total funding for NMRA (in USD) as a proportion to the population in a given year. Secondary data on population from official government sources forms the denominator for the indicator.<br>- **Numerator:** Total annual funding for the NMRA<br>- **Denominator:** Population of the country
**d) Unit of Measurement:** Proportion
**e) Measurement frequency:** Annually
**f) Evidence/ Sources:** CIRS OpERA programme, NMRA records, Ministry of Health
**g) Indicator Classification:** Input
**h) Data Type:** Quantitative
**i) Institutional Responsibility:** NMRA; Ministry of Health
Indicator 6: Reliability of NMRA funding
**a) Purpose:** This indicator assesses the ability of an NMRA to sustain its activities using the revenue generated from various sources and government subvention.
**b) Rationale:** NMRAs funding comes from a number of sources including government subvention, revenue from services rendered and investments as well as donations. The ability of the NMRA to generate and utilise own revenue is critical for strengthening and sustaining its ability to control the market. Furthermore, considering that medical products regulation is a public function, it is anticipated that government substantially supports NMRAs.
**c) Method of measurement:** Indicator 6 is calculated using two methods:<br>1) Financial sustainability measured by assessing trend on annual income over expenditure over study period based on fees for service and government subventions against donor funding.

2)	Total revenue generated by NMRA (in USD) as a percentage of the total NMRA funding[1] (in USD) in a given year. NMRA revenue includes revenue from services rendered and investments.<br>- Numerator Total NMRA revenue generated by NMRA X 100<br>- Denominator Total NMRA funding<br><br>3) Total government[1] funding for NMRA (in USD) as a percentage of the total NMRA funding (in USD) in a given year<br>- Numerator: Total government funding for the NMRA X 100.<br>- Denominator: Total NMRA funding
**d)**	**Evidence/ Sources:** Central Medical Stores financial records, Ministry of finance records, Approved annual national budget, NMRA: CIRS OpERA programme, Central Medical Stores records, Ministry of finance records, Approved annual national budget, NMRA records
**e)**	**Institutional Responsibility:** NMRA; Ministers of Health; Ministry of Finance
**f)**	**Measurement frequency:** Annually
**g)**	**Unit of Measurement:** Percentage (%)
**h)**	**Data Type:** Quantitative
**i)**	**Indicator Classification:** Input
