# Supplementary material for: National medicines regulatory authorities financial sustainability in the East African Community
Source: PLoS One. 2020 Jul 23;15(7):e0236332. doi: 10.1371/journal.pone.0236332 (PMC7377437; doi:10.1371/journal.pone.0236332)
Supplement: S1 Table — (PDF) [file pone.0236332.s003.pdf]

National Medicines Regulatory Authorities Financial Sustainability in the East African Community
Data File

***All figures are in USD**
NA = Data was not provided

**Zanzibar Food and Drugs Authority**						
**Year**	**2011**	**2012**	**2013**	**2014**	**2015**	**2016**
**Budget**	506,172.84	567,901.23	793,209.88	1,104,938.27	1,149,421.12	NA
**Funding and sources**						
Government	123,456.79	135,802.47	123,456.79	308,641.47	571,428.57	NA
Industry Fees	127,916.78	159,646.36	187,866.85	130,550.95	220,535.78	NA
Donors	12,345.68	18,518.52	27,777.78	61,728.40	42,099.75	NA
Others	6,172.84	7,407.41	8,333.33	9,259.26	11,904.76	NA
**Total Funding**	269,892.09	321,374.76	347,434.75	510,180.08	845,968.86	NA
**Total expenditure**	NA	NA	NA	NA	NA	NA

**Tanzania Medicines and Medical Devices Authority (TMDA)**						
**Year**	**2011**	**2012**	**2013**	**2014**	**2015**	**2016**
**Budget**	3,384,123	4,478,192	6,612,822	6,782,285	9,422,888	13,574,502
**Funding and sources**						
Government	481,396.86	1,246,754	1,389,165	1,424,385	1,299,795	2,407,709
Industry Fees	2,018,608.88	3,185,810	4,683,337	5,313,039	8,123,093	11,118,155
Donors	884,118	45,628	540,320	44,861	-	48,639
Others	NA	NA	NA	NA	NA	NA
**Total Funding**	3,384,123	4,478,192	6,612,822	6,782,285	9,422,888	13,574,502
**Total expenditure**	3,384,123	4,478,192	6,612,822	6,782,285	9,422,888	13,574,502

**Kenya Pharmacy and Poisons Board (PPB)**						
**Year**	**2011**	**2012**	**2013**	**2014**	**2015**	**2016**
**Budget**	5,853,000.00	4,366,000.00	7,210,000.00	7,328,824.57	9,009,470.30	11,989,361.82
**Funding and sources**						
Government	NA	NA	NA	NA	NA	NA
Industry Fees	NA	NA	NA	NA	NA	NA
Donors	NA	NA	NA	NA	NA	NA
Others	NA	NA	NA	NA	NA	NA
**Total Funding**	NA	NA	NA	NA	NA	NA
**Total expenditure**	NA	NA	NA	4200211.11	467552.22	503481.11

**Burundi National Medicines Regulatory Agency**						
**Year**	**2011**	**2012**	**2013**	**2014**	**2015**	**2016**
**Budget**	NA	NA	NA	NA	NA	NA
**Funding and sources**						
Government	39,528.89	63,451.85	60,150.50	62,913.62	63,257.57	63,257.57
Industry Fees	NA	NA	NA	NA	NA	NA
Donors	NA	NA	NA	NA	NA	NA
Others	NA	NA	NA	NA	NA	NA
**Total Funding**	39,528.89	63,451.85	60,150.50	62,913.62	63,257.57	63,257.57
**Total expenditure**	38,346.11	63,489.82	60,150.51	62,913.62	2,010.78	2,022.75

**Uganda National Drug Authority (NDA)**						
**Year**	**2011**	**2012**	**2013**	**2014**	**2015**	**2016**
**Budget**	7,119,390.00	10,810,412.00	10,795,264.00	12,657,851.00	10,656,704.00	12,307,598.00
**Funding and sources**						
Government	NA	NA	NA	NA	NA	NA
Industry Fees	8,916,521.00	9,268,816.00	10,653,485.00	11,547,369.00	NA	NA
Donors	133,862.00	396,951.00	141,295.00	150,882.00	NA	NA
Others	NA	NA	NA	NA	NA	NA
**Total Funding**	9,050,383.00	9,665,767.00	10,794,780.00	11,698,251.00	NA	NA
**Total expenditure**	9,050,381.00	9,665,765.00	10,794,779.00	11,698,249.00	NA	NA
